# High Salt Tolerance of a *Bradyrhizobium* Strain and Its Promotion of the Growth of *Stylosanthes guianensis*

**DOI:** 10.3390/ijms18081625

**Published:** 2017-07-28

**Authors:** Rongshu Dong, Jie Zhang, Hengfu Huan, Changjun Bai, Zhijian Chen, Guodao Liu

**Affiliations:** Institute of Tropical Crop Genetic Resources, Chinese Academy of Tropical Agriculture Sciences, Haikou 571101, China; dongrongshu@catas.cn (R.D.); zhangjie@catas.cn (J.Z.); hfhuan@catas.cn (H.H.); baichangjun@catas.cn (C.B.)

**Keywords:** rhizobia, salinity, IAA production, osmoprotectant, proteomics, *Stylosanthes*

## Abstract

Salinity is a serious limiting factor for the growth of rhizobia. Some rhizobia are tolerant to salt stress and promote plant growth, but the mechanisms underlying these effects are poorly characterized. The growth responses and osmoprotectants in four *Bradyrhizobium* strains were examined under salt stress in this study. Two-dimensional electrophoresis (2-DE) and mass spectrometry were conducted to investigate protein profiles in rhizobia exposed to salt stress. Subsequently, salt tolerance in stylo (*Stylosanthes* *guianensis*) inoculated with rhizobia was further detected in hydroponics. Results showed that the *Bradyrhizobium* strain RJS9-2 exhibited higher salt tolerance than the other three *Bradyrhizobium* strains. RJS9-2 was able to grow at 0.35 M NaCl treatment, while the other three *Bradyrhizobium* strains did not grow at 0.1 M NaCl treatment. Salt stress induced IAA production, and accumulation of proline, betaine, ectoine, and trehalose was observed in RJS9-2 but not in PN13-1. Proteomics analysis identified 14 proteins regulated by salt stress in RJS9-2 that were mainly related to the ABC transporter, stress response, and protein metabolism. Furthermore, under saline conditions, the nodule number, plant dry weight, and N concentration in stylo plants inoculated with RJS9-2 were higher than those in plants inoculated with PN13-1. These results suggest that the tolerance of RJS9-2 to salt stress may be achieved by the coordination of indole-3-acetic acid (IAA) production, osmoprotectant accumulation, and protein expression, thus promoting stylo growth.

## 1. Introduction

Rhizobia are important soil bacteria that fix atmospheric N_2_ in symbiotic root nodules and thus supply legume plants with nitrogen (N) [1]. However, rhizobial strains are sensitive to environmental factors, such as salt, temperature, and pH stresses, which affect their symbiotic N fixation (NSF) capacity and thus limit legume productivity [2]. To date, more than 800 million hectares of land globally, representing 40% of the world’s land surface, are affected by salinity, which is an important factor limiting the growth of rhizosphere rhizobia [3,4]. Salinity has detrimental effects on the activity of rhizobia, such as decreasing the rhizobial cell number and restricting root infection, accompanied by inhibiting nodule development and impairing nodule activity [2,5,6].

Rhizobial growth is affected by NaCl treatment, and different rhizobial strains exhibit significant differences in salt tolerance capability [2,6]. For example, the *Rhizobium japonicum* strain USDA191 is more tolerant to 0.4 M NaCl treatment than is the strain USDA110 [7]. *Mesorhizobium* strains are more sensitive to salt stress than are strains of *Rhizobium* and *Sinorhizobium* but are more tolerant to salt stress than are strains of *Bradyrhizobium* [2,5]. Therefore, the isolation of novel salt-tolerant rhizobia may be one of the most valuable methods by which to alleviate the environmental problem of salinity and thus favor the growth of host plants under salinity [8,9]. Furthermore, elucidating the physiological and molecular mechanisms underlying the rhizobia in responses to salt stress is vital for improving rhizobial strains with a highly efficient symbiotic capability.

Rhizobia develop specific strategies to cope with salt stress, including the accumulation of inorganic cations (e.g., potassium) and the intracellular production of low-molecular-weight organic solutes, such as proline, trehalose, glycine betaine, ectoines, and polyamines. These compounds protect cells from desiccation and osmotic stress by stabilizing the conformation of proteins and biological membranes [10,11,12,13]. For example, proline is a highly soluble neutral compound that is regarded as an osmoprotectant involved in the bacterial response to osmotic stress [10]. Compounds structurally related to betaines, such as glycine betaine, proline betaine, and carnitine, play a major role in osmoregulation in rhizobial bacteria under salt stress, such as in *R. meliloti*, *Rhizobium* sp., *Mesorhizobium* sp., and *S. meliloti* [12,14,15,16]. Furthermore, trehalose is an important osmoprotectant for increasing osmotolerance in *S*. *meliloti* [17].

In root nodules fixing N_2_ symbiotically, not only the bacteria but also the host plants are sensitive to salinity. The symbiotic root nodules are more sensitive to NaCl exposure than are free-living rhizobia [8,9,18]. Nodulation, nitrogen fixation, and plant growth are inhibited by salt stress, whereas the inoculation of legumes, including soybean (*Glycine max*), common bean (*Phaseolus vulgaris*), and alfalfa (*Medicago sativa*), with salt-tolerant rhizobia is beneficial for host plant growth under saline conditions [8,9,18,19]. For example, the growth of alfalfa is enhanced when inoculated with the salt-tolerant *Rhizobium* strain Rm1521 rather than with the salt-sensitive strain A2 under NaCl treatments [8].

Stylo (*Stylosanthes* spp.) is an important forage legume that is grown in tropical and subtropical areas [20,21]. Stylo is mainly used in agricultural systems for livestock nutrition, also for soil improvement, sown pastures, improving natural grassland, and orchard mulching [20]. A large number of stylo germplasm has been introduced to China from Australia and Colombia since the 1960s, and stylo has become the most important tropical forage legume widely cultivated in South China [22]. Stylo, similar to other legumes that form symbioses with rhizobia to form root nodules, has a low yield when under salinity stress [23,24]. Furthermore, stylo species exhibit different capability of salt stress tolerance. *S. guianensis* displays higher seed germination rates and higher growth and survival rates than *S. humilis* and *S. capitata* under 0.15 M NaCl treatment [23]. In a previous study, we isolated a variety of salt-tolerant *Bradyrhizobium* strains belonging to the genus *Bradyrhizobium* from nodules of stylo plants grown in different habitats across China [25]. Since the salt-tolerant *Bradyrhizobium* strains may also act as an efficient bioinoculant for stylo, we hypothesize that these strains may promote the growth of stylo under saline conditions through their higher tolerance to salt. However, the specific physiological and molecular mechanisms underlying the adaptation of these rhizobia to salt stress remain unknown. In this study, the effects of salt stress on the growth and responses of four *Bradyrhizobium* strains were examined. Two-dimensional electrophoresis (2-DE) combined with matrix-assisted laser desorption/ionization time of flight mass spectrometry (MALDI-TOF-MS) was used to investigate the protein profiles in rhizobial cells exposed to salt stress. The effects of rhizobial inoculation on osmotolerance in stylo under salt stress were then examined.

## 2. Results

### 2.1. Variability of Salt Response in Bradyrhizobium Strains

After isolation from stylo nodules, four *Bradyrhizobium* strains, BS1-1, PN13-3, LZ3-2, and RJS9-2, were inoculated onto solid YMA medium supplied with NaCl at concentrations ranging from 0.0017 (CK) to 0.5 M (Figure S1). After 6 days of incubation, the rhizobial growth of all strains was obviously affected by salt stress but showed variation among different strains (Figure S1, Table 1). The growth of BS1-1, PN13-3, and LZ3-2 was significantly inhibited by NaCl at up to 0.1 M. The salt-tolerant strain RJS9-2 showed higher salt tolerance than the other three salt-sensitive strains, as reflected by better growth performances under 0.35 M NaCl treatments. No significant differences in growth performance were observed among the four tested strains under control conditions (Table 1).

**Figure 1:**
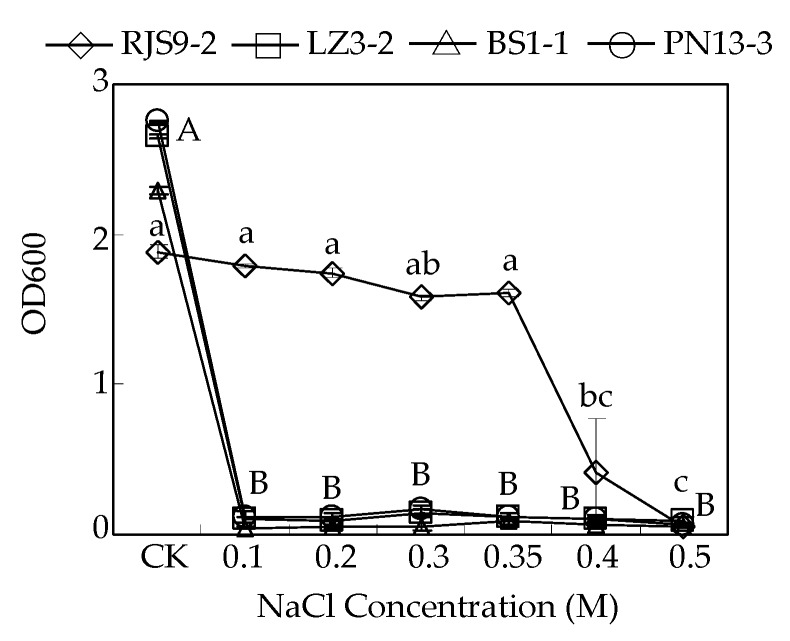
Effects of different NaCl levels on the growth of rhizobia. The four tested *Bradyrhizobium* strains were separately inoculated onto liquid YMA medium containing 0.0017 (CK), 0.1, 0.2, 0.3, 0.35, 0.4, or 0.5 M NaCl and further incubated at 28 °C and 180 rpm. The optical density (OD) of the cultures was recorded at 600 nm after 8 days of NaCl treatment. Each bar represents the mean of four biological replicates with standard error. An individual flask was considered one biological replicate. The same lowercase letter indicates no significant difference at *p* < 0.05 (Duncan’s test) in RJS9-2 among different treatments. The same uppercase letter indicates no significant difference at *p* < 0.05 (Duncan’s test) in LZ3-2, BS1-1, and PN13-3 among different treatments, respectively. Due to the same results from Duncan’s test of LZ3-2, BS1-1, and PN13-3, only one group of representative uppercase letters is showed in the figure.

As shown in Figure 1, the growth of the salt-sensitive strains, BS1-1, PN13-3, and LZ3-2, was significantly inhibited by more than 95% under 0.1 M NaCl treatment compared to their respective controls. The salt-tolerant *Bradyrhizobium* strain RJS9-2 could still grow at 0.35 M NaCl treatment (Figure 1). However, the growth of RJS9-2 was inhibited severely by nearly 80% and 100% only under treatments of 0.4 M and 0.5 M NaCl, respectively, compared to bacterial growth under control conditions (Figure 1).

**Figure 2:**
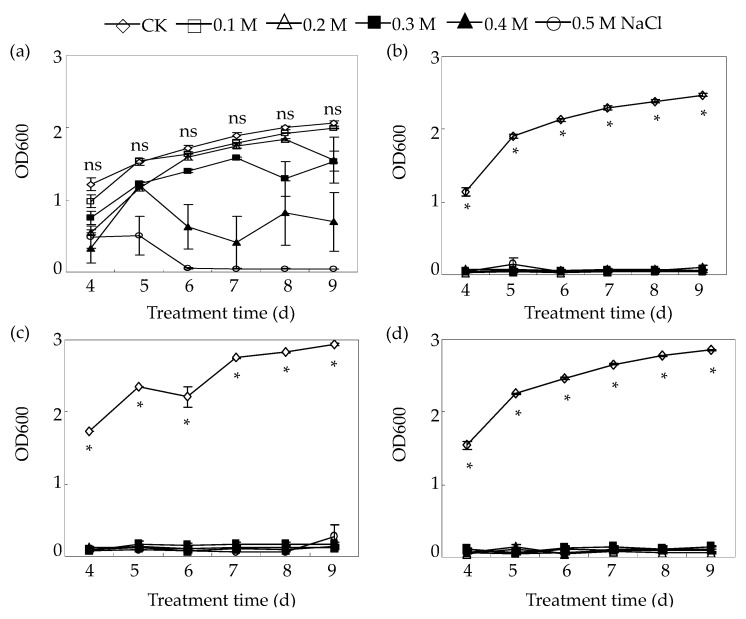
Rhizobial growth affected by NaCl treatment. (**a**) RJS9-2; (**b**) BS1-1; (**c**) PN13-3; (**d**) LZ3-2. The four tested *Bradyrhizobium* strains were separately inoculated into liquid medium containing 0.0017 (CK), 0.1, 0.2, 0.3, 0.4, or 0.5 M NaCl and incubated at 28 °C for 4 to 9 days. The optical density (OD) of the cultures was recorded every 24 h at 600 nm. Each bar represents the mean of four biological replicates with standard error. An individual flask was considered one biological replicate. Asterisks (*) indicate significant differences between CK and other NaCl concentrations at a given time at *p* < 0.05 (Student’s *t*-test). ns indicates no significant difference between CK and other NaCl concentrations at a given time at *p* < 0.05 (Student’s *t*-test).

The growth responses to salt stress among the four *Bradyrhizobium* strains were further examined under different NaCl treatments during the incubation period from 4 to 9 days (Figure 2). The different responses of the four tested strains to salt stress were observed (Figure 2). The growth of the salt-tolerant strain RJS9-2 was not inhibited under NaCl treatment at 0.1 to 0.3 M for 4 to 9 days, although it was inhibited under NaCl treatment at a concentration greater than 0.3 M (Figure 2a). However, the growth of the salt-sensitive strains, BS1-1, PN13-3, and LZ3-2, was inhibited significantly by 4 days under 0.1 M NaCl or above compared to the respective controls (Figure 2b–d). Overall, these results demonstrate that RJS9-2 is more tolerant to salt than are the other three *Bradyrhizobium* strains.

### 2.2. Changes in Indole-3-Acetic Acid (IAA) Production in Response to Salt Stress

**Figure 3:**
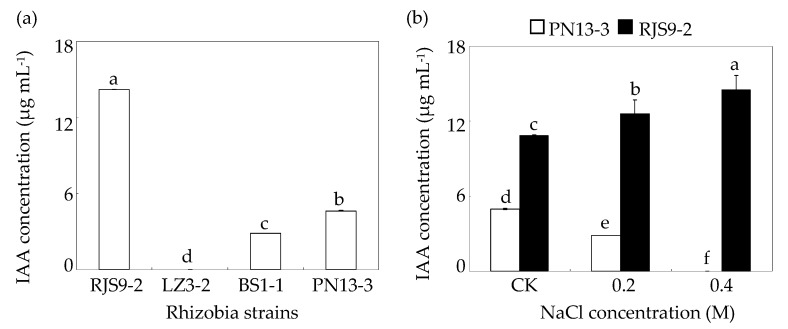
Indole-3-acetic acid (IAA) concentrations in rhizobia at various NaCl levels. (**a**) IAA concentrations in the four tested strains in the absence of NaCl treatment; (**b**) IAA concentrations in PN13-3 and RJS9-2 under various NaCl concentrations. *Bradyrhizobium* strains were separately inoculated into liquid medium containing 0.0017 (CK), 0.2, or 0.4 M NaCl and further incubated at 28 °C and 180 rpm. The IAA concentrations in the culture medium were detected after 8 days of NaCl treatment. A column represents mean value obtained from three biological replicates and bar represents standard error. An individual flask was considered one biological replicate. The same letter indicates no significant difference at *p* < 0.05 (Duncan’s test).

As shown in Figure 3a, the salt-tolerant strain RJS9-2 produced the highest amount of IAA among all of the *Bradyrhizobium* strains; in contrast, LZ3-2 did not produce IAA under control conditions. The IAA concentrations in the liquid growth medium of RJS9-2 were 5.0-fold and 3.0-fold higher than those in BS1-1 and PN13-3, respectively (Figure 3a). Furthermore, IAA productions differed between PN13-3 and RJS9-2 and changed in response to NaCl supply (Figure 3b). Increasing IAA concentrations in response to salt stress were observed in the salt-tolerant strain RJS9-2 but not in the salt-sensitive strain PN13-3 (Figure 3b). The IAA concentration in RJS9-2 significantly increased by about 20% and 30% under 0.2 and 0.4 M NaCl treatments compared to the control treatments, respectively (Figure 3b). However, 0.2 M NaCl treatment obviously decreased the IAA concentration in PN13-3, and 0.4 M NaCl totally inhibited IAA production in PN13-3. Moreover, IAA concentration in the salt-tolerant strain RJS9-2 was 4.0 times higher than that in the salt-sensitive strain PN13-3 under 0.2 M NaCl treatment (Figure 3b). These results indicate that salt stress induces IAA secretion only in the salt-tolerant strain RJS9-2.

### 2.3. Responses of Osmoprotectants to Salt Stress

**Figure 4:**
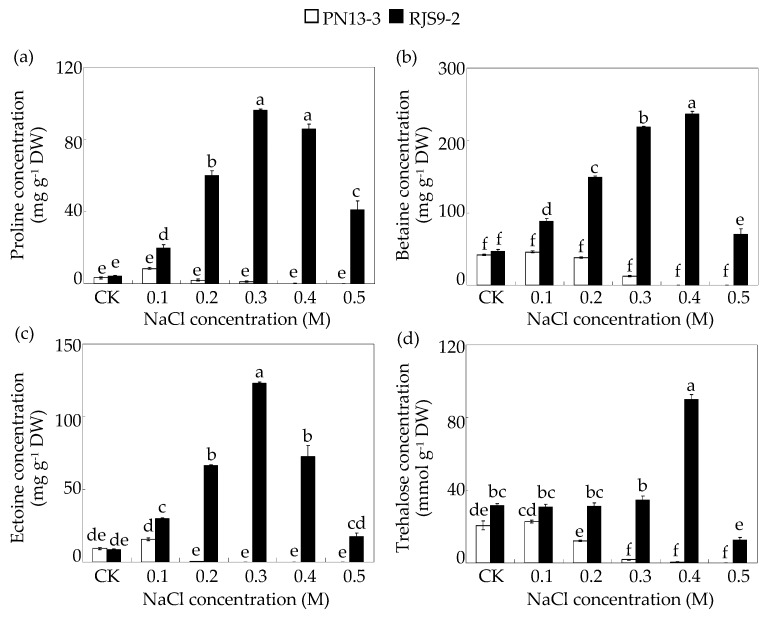
Osmoprotectant concentrations in two *Bradyrhizobium* strains at various NaCl levels. (**a**) Proline concentrations; (**b**) Betaine concentrations; (**c**) Ectoine concentrations; (**d**) Trehalose concentrations. *Bradyrhizobium* strains were separately inoculated into liquid medium containing 0.0017 (CK), 0.1, 0.2, 0.3, 0.4, or 0.5 M NaCl and further incubated at 28 °C and 180 rpm. The osmoprotectant concentrations in the bacteria were detected after 8 days of NaCl treatment. A column represents mean value obtained from four biological replicates and bar represents standard error. An individual flask was considered one biological replicate. The same letter indicates no significant difference at *p* < 0.05 (Duncan’s test).

The concentrations of four osmoprotectants, proline, betaine, ectoine, and trehalose, were further detected in PN13-3 and RJS9-2 under NaCl treatments. As shown in Figure 4, the responses of the four tested osmoprotectants to increased NaCl supply varied greatly between PN13-3 and RJS9-2. Proline, betaine, and ectoine concentrations in the salt-tolerant strain RJS9-2 but not the salt-sensitive strain PN13-3 increased with increasing NaCl levels and reached a maximum at 0.3 or 0.4 M NaCl (Figure 4a–c). The concentrations of proline, betaine, and ectoine in RJS9-2 increased by nearly 20-fold, 10-fold, and 4-fold under 0.3 M NaCl treatments compared to the control treatments (CK), respectively (Figure 4a–c). Furthermore, the trehalose concentration in RJS9-2 increased only under 0.4 M NaCl treatment compared to CK (Figure 4d). However, the concentrations of four tested osmoprotectants in PN13-3 decreased as NaCl supply increased, especially for the NaCl concentration up to 0.2 or 0.3 M. Furthermore, across all NaCl levels, the salt-tolerant strain RJS9-2 exhibited the higher osmoprotectant concentrations than the salt-sensitive strain PN13-3 (Figure 4). The concentrations of the four tested osmoprotectants in the salt-tolerant strain RJS9-2 were more than 200 times greater than those in the salt-sensitive strain PN13-3 under 0.4 M NaCl treatment (Figure 4).

### 2.4. Identification of Salt-Stress-Responsive Proteins

**Figure 5:**
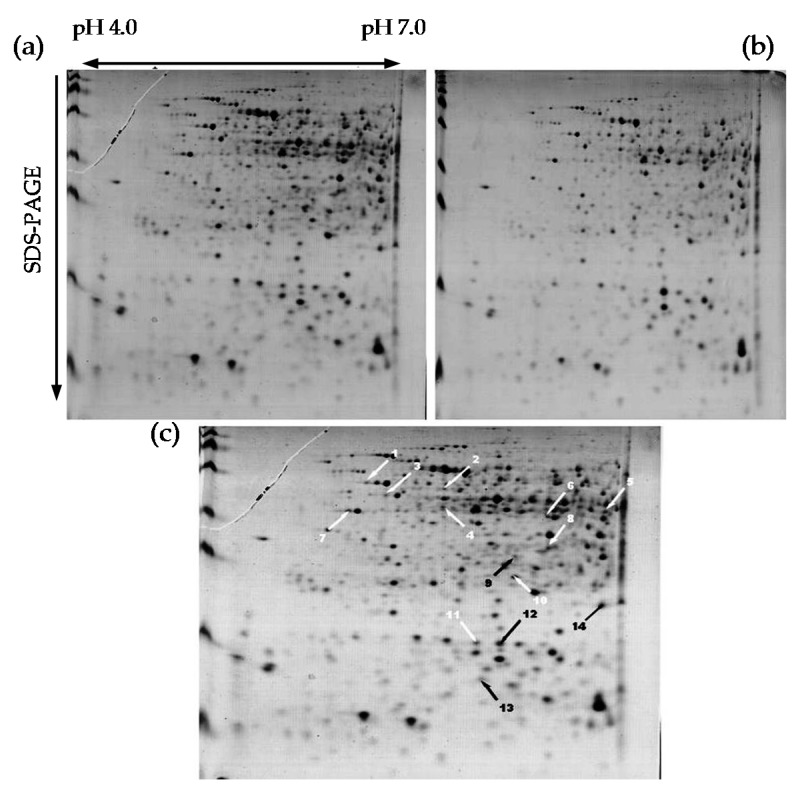
Protein profile of RJS9-2 at two NaCl levels through 2-DE analysis. (**a**) Protein profiles in the 0.0017 M NaCl treatment; (**b**) Protein profiles in the 0.3 M NaCl treatment; (**c**) Picture of two merged 2-DE gels under 0.0017 and 0.3 M NaCl treatments. After bacterial cell proteins were extracted, 80 µg of protein was loaded onto an IPG strip (pH 4–7, 11 cm) and separated by SDS-PAGE. Protein spots showing enhanced and repressed accumulation between the 0.0017 M and 0.3 M NaCl treatments are highlighted with black and white arrows, respectively.

To identify differentially expressed proteins regulated by salt stress in the salt-tolerant strain RJS9-2, two-dimensional electrophoresis (2-DE) combined with MALDI TOF/TOF MS was conducted to identify salt stress responsive proteins in the salt-tolerant strain RJS9-2 under control (0.0017 M NaCl) and 0.3 M NaCl treatments. Approximately 400 protein spots were effectively separated in the 2-DE gels (Figure 5). A total of fourteen protein spots with an average abundance change of more than 1.5-fold were found to display differential accumulation between the control and 0.3 M NaCl treatments (Figure 5). Subsequently, these protein spots were successfully identified by MALDI TOF/TOF MS analysis (Table 2, Table S1).

Among the identified proteins, accumulations of four proteins were increased by salt stress. These proteins were classified into four functional groups, including carbon metabolism, stress response, transporter, and signal transduction, each with one protein (Table 2). In addition, the ten proteins repressed by salt stress were separated into five functional groups, including protein metabolism with four proteins, energy metabolism and other metabolism with two proteins each, and transporter and signal transduction with one protein each (Table 2).

### 2.5. Stylo Growth as Affected by Rhizobial Inoculation and Salt Stress

**Figure 6:**
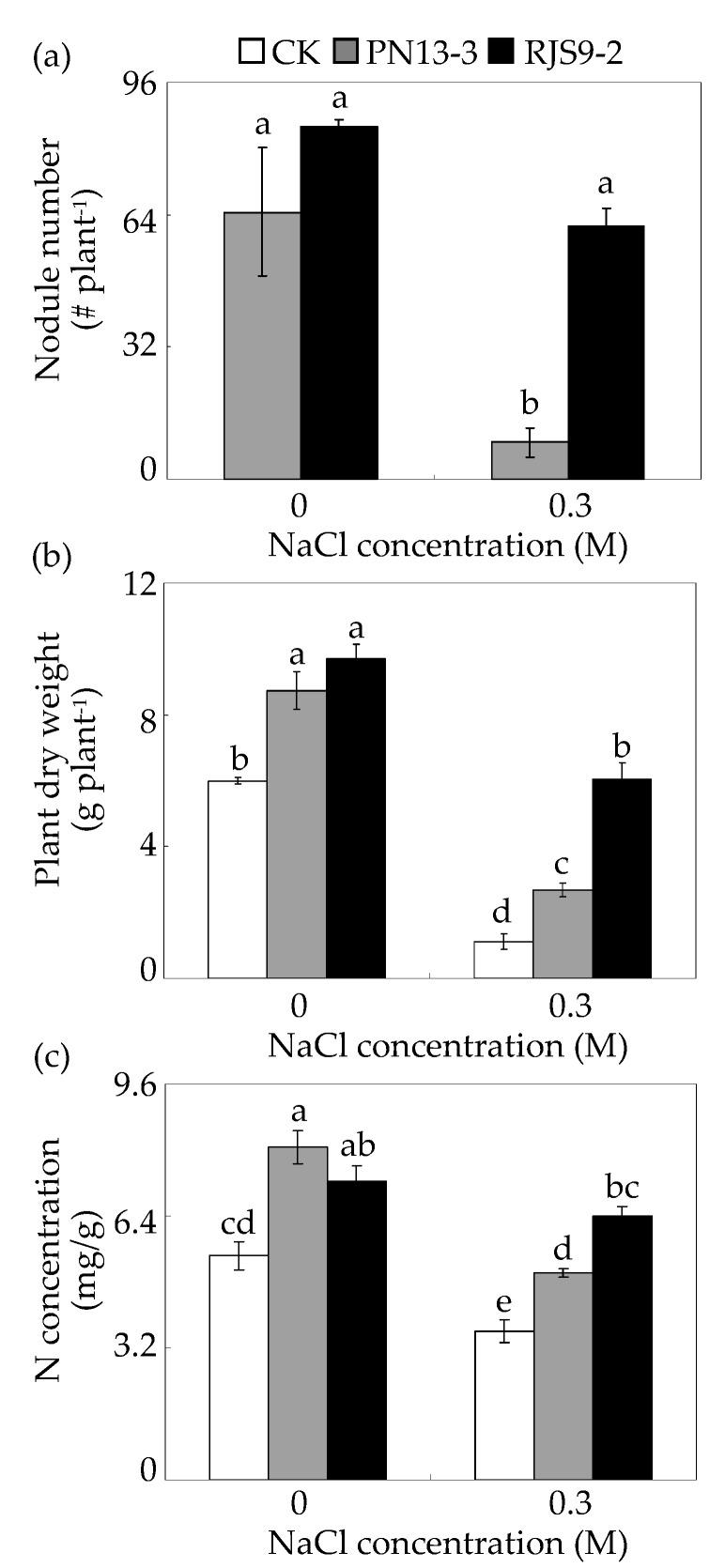
Plant and nodule growth affected by rhizobial inoculation and salt stress. (**a**) Nodule number; (**b**) Plant dry weight; (**c**) N concentration. Twenty-four-day-old seedlings without (CK) or with rhizobial inoculation were transplanted into nutrient solution under 0 or 0.3 M NaCl treatment. The shoots, roots, and nodules were harvested separately after 40 days of NaCl treatments. A column represents mean value obtained from three biological replicates and bar represents standard error. An individual hydroponics box contained three seedlings was regarded as one biological replicate. The same letter indicates no significant difference at *p* < 0.05 (Duncan’s test). # indicates the number of nodule.

Subsequently, the effects of rhizobial inoculation on the growth of stylo subjected to salt stress were further examined. The results showed that nodule and plant growth were affected by rhizobial inoculation and salt stress (Figure 6). The nodule number in stylo plants inoculated with PN13-3 significantly decreased by nearly 90% under 0.3 M NaCl treatment compared to without NaCl treatment (Figure 6a). Furthermore, the nodule number was about 600% higher in stylo plants inoculated with the salt-tolerant strain RJS9-2 than in those inoculated with the salt-sensitive strain PN13-3 under 0.3 M NaCl treatment (Figure 6a). The plant dry weight was obviously decreased by NaCl treatment with or without rhizobial inoculation, but differed in inoculant treatments. Although the plant dry weight obviously decreased by salt stress, the plant dry weight was increased by more than 100% and 400% with PN13-3 and RJS9-2 inoculation compared to that without rhizobial inoculation under 0.3 M NaCl treatment, respectively (Figure 6b). Interestingly, the plant dry weight was about 100% higher in stylo plants inoculated with the salt-tolerant strain RJS9-2 than in plants inoculated with the salt-sensitive strain PN13-3 under 0.3 M NaCl treatment (Figure 6b).

The N concentrations in plants increased upon inoculation with either rhizobial strain, with or without NaCl treatments (Figure 6c). Furthermore, the N concentration in plants inoculated with the salt-tolerant strain RJS9-2 was 30% higher than that in plants inoculated with the salt-sensitive strain PN13-3 under 0.3 M NaCl treatment (Figure 6c). These results suggest that under salt stress, the symbiotic efficiency is greater in stylo inoculated with the salt-tolerant strain RJS9-2 compared to that in stylo inoculated with PN13-3.

## 3. Discussion

Salinity is one of the most serious environmental stresses limiting the growth of rhizobia, which fix atmospheric N_2_ and supply host plants with N [6,9]. Cumulative results suggest that rhizobia show different levels of tolerance to salt stresses [2,5,26]. Most *Rhizobium* strains that are used as standards do not survive under about 0.2 M NaCl, except for strain USDA 2671, which can grow under 0.8 M NaCl but is inhibited by treatment with 1 M NaCl [26]. Furthermore, among the rhizobial strains belonging to *Mesorhizobium*, *Sinorhizobium*, and *Rhizobium*, *S. meliloti* strains are the most tolerant and can grow under 0.8 M NaCl, while the growth of *R. gallicum* and *M. mediterraneum* is inhibited by 0.2 M NaCl treatment [27]. In general, strains of bradyrhizobia are considered as salt-sensitive strains compared to the other rhizobial strains. Among the 190 *Brudyrlzizobiuni* strains, 40 strains grow well at 0.2 M NaCl treatment while only 12 strains survive under 0.4 M NaCl condition [28]. For example, *B. juponicurn* strain A1017 was completely inhibited by 0.15 M NaCl [29]. Thus, the tolerance of rhizobia to salinity is considered to be more strain-specific than species-specific [30]. In this study, the salt-tolerant *Bradyrhizobium* strain RJS9-2 was able to grow at 0.35 M NaCl treatment, while the growth of the other three salt-sensitive *Bradyrhizobium* strains was significantly inhibited under 0.1 M NaCl (Table 1, Figure 1 and Figure 2). Therefore, among these *Bradyrhizobium* strains isolated from stylo plants, RJS9-2 shows greater salt tolerance.

As a ubiquitous molecule, indole-3-acetic acid (IAA) plays important roles in many living organisms against stress conditions probably through maintaining endogenous hormone level and enhancing different cellular defense systems, and IAA production by rhizobial isolates has been demonstrated by many studies, such as in *Rhizobium* and *Bradyrhizobium* strains [1,31,32]. It has been demonstrated that IAA enhances protection of bacteria cells against abiotic stresses, including salinity and oxidative stresses [33]. In this study, IAA analysis showed that under normal growth conditions, the IAA concentrations in the liquid growth medium of the salt-tolerant strain RJS9-2 were significantly higher than those in the other three salt-sensitive strains, although most of the *Bradyrhizobium* strains were capable of producing IAA (Figure 3a). Furthermore, salt-induced IAA secretion was observed in the salt-tolerant strain RJS9-2 but not in the salt-sensitive strain PN13-3 (Figure 3b). Consistent with our results, *R. phaseoli* strains isolated from mung bean (*Vigna radiata*) nodules produce IAA, and the highest IAA-producing rhizobial isolate, N20, exhibits salt tolerance compared to the other rhizobial strains with a low level of IAA production [34]. Furthermore, inoculation with salt-tolerant, auxin-producing bacteria has been reported to promote the growth of plants under salt-stressed conditions [34,35,36]. Therefore, increasing IAA production in the salt-tolerant strain RJS9-2 might increase its salt tolerance.

The accumulation of osmoprotective compounds by de novo biosynthesis in rhizobia is considered an effective strategy of adaptation by rhizobia to salt stress by protecting cells from desiccation and osmotic stress [6,11,12]. Furthermore, application of exogenous osmoprotective compounds increases the salt tolerance of rhizobial strains, such as *Mesorhizobium* sp. (USDA3350 and ACMP18), indicating these molecules involve in regulation of osmotic balance [12]. In this study, compared to control conditions, the concentrations of proline, betaine, and ectoine in the salt-tolerant strain RJS9-2 significantly increased under all NaCl levels, and the trehalose concentrations in RJS9-2 increased under 0.4 M NaCl (Figure 4). However, the concentrations of the four tested osmoprotectants in the salt-sensitive strain PN13-3 were significantly inhibited by salt stress and were also lower than those in the salt-tolerant strain RJS9-2 (Figure 4). It has been showed that under salt stress, the growth of *B. japonicum* strain RCR 3827 is enhanced when trehalose was added into the medium, suggesting that trehalose may act as an osmoregulator in *Bradyrhizobium* strains in response to salt stress [37]. These results are also consistent with those from other rhizobia, such as *M. ciceri* and *S. meliloti* [6,16] suggesting that the ability to accumulate osmoprotectants in the salt-tolerant rhizobial strain RJS9-2 may be beneficial to bacterial growth through reducing osmotic stress caused by salts. However, the effects of proline, ectoine, and glycine betaine on the growth of rhizobia under salt stress do not seem to be general or to directly contribute to growth enhancement, which might depend on the rhizobial species [14,38,39]. Thus, the exact roles of osmoprotective compounds in the salt tolerance of RJS9-2 merit further study.

In this study, a proteomic approach was used to identify salt-stress-responsive proteins in the salt-tolerant strain RJS9-2 under control and 0.3 M NaCl treatments. A total of 14 protein spots exhibiting differential responses to salt stress were successfully identified (Figure 5, Table 2), suggesting that the putative functions and pathways associated with the identified proteins might be involved in the response to salt stress in rhizobia. Two transporter proteins, ABC transporter and branched-chain amino acid ABC transporter substrate-binding protein (spots 3 and 13), were associated with ABC transporter systems in RJS9-2 and were regulated by salt stress in the current study (Table 2). ABC transporter proteins play important roles in the transportation of nutrients and small molecules are involved in cellular osmoprotection and contribute to bacterial adaptation to hyperosmolarity. ABC transporters are regulated by salt stress in many rhizobial strains, such as *S. meliloti*, *R. etli*, *M. ciceri*, and *R. tropici* [40,41,42,43]. For example, salt stress induces the accumulation of a putative oligopeptide ABC transporter ATP-binding protein in the halotolerant strain A5. The putative oligopeptide ABC transporter ATP-binding protein may be involved in the transportation of small molecules (e.g., amino acids, amines, and peptides), and it plays a key role in the modulation of solute diffusion under osmotic stress in strain A5 [42]. Therefore, the regulation of ABC transporter systems combined with the de novo biosynthesis of an osmoprotectant might be a potential adaptive strategy of the salt-tolerant strain RJS9-2 in response to high salinity.

Rhizobia develop particular biochemical mechanisms to overcome severe osmotic conditions, such as the regulation of the enzymatic antioxidant system [44,45]. Previous studies have suggested that bacterial superoxide dismutase (SOD) plays a critical role in scavenging reactive oxygen species and protecting the nitrogen fixation process in response to osmotic stress [44,45]. Salt stress increases the SOD activity in strains of both *S. meliloti* and *Mesorhizobium*, especially for the most salt-tolerant strain of *S. meliloti*, 4H41 [27]. Furthermore, the expression of the *SOD* gene is induced in *S. meliloti* in response to salt stress [45]. In this study, the accumulation of SOD (spot 2) was increased by salt stress in RJS9-2 (Table 2), suggesting that the increasing accumulation of SOD in the salt-tolerant strain RJS9-2 might help to prevent structural and functional damages caused by osmotic stress.

In the present study, accumulations of four proteins related to protein metabolism were repressed by salt stress, including zinc protease, elongation factor Ts, and the 30S ribosomal protein S6 (spots 5–8; Table 2). Consistent with our findings, metabolism-related proteins are regulated by salt stress at the protein or transcriptional level [42,45,46]. Most of the salt-repressed genes encoding proteins, such as ribosomal proteins and elongation factors, in *S. meliloti* are involved in metabolism, suggesting that protein synthesis is probably inhibited under salt stress [45]. Furthermore, in this study, the accumulation of energy-related proteins, such as ATP synthase subunit beta and enolase, was repressed by salt stress (spots 9 and 10; Table 2). The subunits of energy-production proteins are regulated by salt stress in *Sinorhizobium* sp. BL3 and might function in maintaining the energy balance [41]. Therefore, metabolism-related proteins regulated by salt stress in the salt-tolerant strain RJS9-2 might play important roles in adaptation to salt stress. However, the precise functions of these proteins require further study, and support of our conclusions requires additional data.

Rhizobial symbiosis increases salt-stress tolerance in legumes grown under saline conditions [8,9]. It has recently been reported that the rhizobial strain *S. meliloti* 1021 has the ability to enhance salt stress tolerance in soybean, possibly by decreasing ionic stress, increasing the antioxidant enzyme activity and the content of osmotic compounds, and regulating the expression of key genes involved in reactive oxygen species (ROS) scavenging (e.g., *SOD*) [9]. Furthermore, rhizobial inoculation, particularly for salt-tolerant rhizobial strains, enhances the host plant’s tolerance to salt stress. For example, compared to the salt-sensitive *Rhizobium* strain A2, the growth of alfalfa in the presence of NaCl is promoted with inoculation of the salt-tolerant strain Rm1521 [8]. In this study, both RJS9-2 and PN13-3 effectively promoted plant growth and N fixation under control conditions (Figure 6). However, compared to the salt-sensitive strain PN13-3, inoculation with the salt-tolerant strain RJS9-2 was more effective at enhancing the tolerance of stylo to salt stress, as reflected by increases in nodule numbers, plant dry weight, and N concentrations (Figure 6). Therefore, increasing the salt tolerance of stylo might be due to either RJS9-2’s effective production of IAA helping the stylo plant to maintain hormone level, regulating plant cellular processes and symbiotic N fixation capability, or increasing the content of osmoprotective compounds (e.g., proline, ectoine, and glycine betaine), reducing osmotic stress caused by salts.

## 4. Materials and Methods

### 4.1. Bradyrhizobium Strains and Growth Conditions

In this study, four *Bradyrhizobium* strains, BS1-1, PN13-3, LZ3-2, and RJS9-2, were used. BS1-1, PN13-3, LZ3-2, and RJS9-2 were originally isolated and purified from the nodules of stylo plants grown at the Baise, Panzhihua, Liuzhou, and Baoshan field sites in China, respectively. Among these strains, based on morphological and 16S rDNA sequence detection analysis, RJS9-2 belongs to *Bradyrhizobium yuanmingense*, while BS1-1 and LZ3-2 belong to *Bradyrhizobium* sp. PAC40, and PN13-3 belongs to *Bradyrhizobium* sp. Wall9 [25].

The four tested *Bradyrhizobium* strains were grown on solid yeast mannitol agar (YMA) medium containing 10 g·L^−1^ mannitol, 0.2 g·L^−1^ MgSO_4_·7H_2_O, 0.1 g·L^−1^ (0.0017 M) NaCl, 0.25 g·L^−1^ K_2_HPO_4_, 0.25 g·L^−1^ KH_2_PO_4_, and 15 g·L^−1^ agar (pH 6.8). For NaCl treatments, a single rhizobium clone of one of the four strains was inoculated onto solid medium supplied with 0.0017, 0.1, 0.2, 0.3, 0.35, 0.4, or 0.5 M NaCl. Among different NaCl concentrations, 0.0017 M NaCl was set as the control (CK). All of the plates were incubated in an incubator at 28 °C in the dark. Each treatment had four biological replicates. An individual plate was considered one biological replicate. After 6 days of NaCl treatments, the growth performances of the four tested strains were photographed and recorded. The growth activity of the four strains was classified into five groups according to the responses of the strains to salt stress, as follows: ++++, no inhibition by salt stress; +++, 30% growth inhibition by salt stress; ++, 60% growth inhibition by salt stress; +, 90% growth inhibition by salt stress; and −, no growth under salt stress.

The four tested *Bradyrhizobium* strains were separately inoculated onto 50 mL of liquid YMA medium in the flasks. Flasks containing the various strains were incubated on a rotary shaker at 28 °C and 180 rpm until the absorbance reached 0.6 at 600 nm. Subsequently, 1 mL of each culture was added to 100 mL of fresh liquid medium in a flask containing different concentrations of NaCl as described above, and then the flasks were further incubated on a rotary shaker at 28 °C and 180 rpm. The optical density (OD) of the cultures was recorded every 24 h at 600 nm. Each treatment had four biological replicates. An individual flask was considered one biological replicate.

### 4.2. Indole-3-Acetic Acid (IAA) Determination

For each rhizobium, 2 mL samples of cells cultured for 8 days in 100 mL of liquid medium in a flask with each of the various NaCl treatments were centrifuged at 10,000× *g* for 10 min. The supernatant was collected and extracted with an IAA detection kit containing acetate and ethyl acetate (Comin, Suzhou, China). After drying in nitrogen, the remaining precipitated pellets were dissolved in 0.3 mL methanol. All samples were passed through 0.22 μm filters and analyzed by HPLC (L3000, Rigol, Beijing, China) according to the protocol. A standard curve was used to quantify the IAA concentrations in each sample. Each treatment had three biological replicates. An individual flask was considered one biological replicate.

### 4.3. Detection of Osmoprotective Compounds

The four *Bradyrhizobium* strains were separately inoculated into 50 mL of liquid medium in the flasks and then incubated on a rotary shaker at 28 °C and 180 rpm until the absorbance reached 0.6 at 600 nm. Subsequently, 1 mL of each culture was added to 100 mL of fresh liquid medium containing one of the various concentrations of NaCl, and the flasks were then further incubated on a rotary shaker at 28 °C and 180 rpm for 8 days.

For proline determination, 50 mL of the bacterial cultures incubated at 28 °C for 8 days was collected by centrifugation at 6000× *g* for 15 min at 4 °C. The precipitated pellets were dissolved in 5 mL of 3% (*w/v*) sulfosalicylic acid at 100 °C for 10 min. Then, 5 mL of the extracts was added to an equal volume (5 mL) of 2.5% (*w/v*) acid ninhydrin colorimetric buffer containing 2.4 M acetate. After incubation at 100 °C for 45 min, 5 mL of toluene was added to the mixtures. The proline concentration was spectrophotometrically detected at 515 nm according to Pesci and Beffagna [47]. A standard curve was used to quantify the proline concentration in each sample.

To analyze the ectoine concentration, 50 mL of the bacterial cultures incubated at 28 °C for 8 days was centrifuged at 12,000× *g* for 30 min at 4 °C and lyophilized. The precipitated cells were dissolved in 400 μL of methanol:chloroform:water (10:5:4, *v/v/v*) solution by vigorous shaking for 60 min. Equal volumes (130 μL) of chloroform and water were added to the mixtures. After vigorous mixing, the samples were centrifuged at 12,000× *g* for 25 min at 4 °C. The water phase was recovered and lyophilized. The pellet was resuspended in 100 μL of distilled water and 400 μL of acetonitrile (ACN). The ectoine concentration was measured by HPLC (1260 Infinity LC, Agilent, Palo Alto, CA, USA) as described by Anne and Erhard [48].

Betaine was spectrophotometrically detected according to published methods [49] with some modification. Briefly, 50 mL of the bacterial cultures incubated at 28 °C for 8 days was centrifuged at 6000× *g* for 15 min at 4 °C. The precipitated pellets were dissolved in a mixture of methanol:chloroform: 0.2 M KHCO_3_ (12:5:1, *v/v/v*) solution, and the mixture was centrifuged at 10,000× *g* for 15 min at 4 °C. The supernatants were collected and combined with 1 mL of 70% ethanol. After reacting at 65 °C for 5 min, the mixture was centrifuged at 10,000× *g* for 15 min at 4 °C, and then the supernatants were freeze-dried and dissolved in 3 mL of distilled water, followed by incubation with 5 mL of 1.5% (*w/v*) Reinecke salt solution for 1 h at 4 °C. After centrifugation at 10,000× *g* for 15 min at 4 °C, the pellets were resuspended in 5 mL of 99% ether solution, and the mixtures were centrifuged at 10,000× *g* for 15 min at 4 °C. The pellets were vacuum-dried and dissolved in 5 mL of 70% acetone. After reacting for 5 min, the betaine content was determined according to the absorbance at 525 nm.

For trehalose determination, 50 mL of the bacterial cultures incubated at 28 °C for 8 days was harvested by centrifuging at 12,000× *g* for 15 min at 4 °C. The cells were washed with distilled water and then extracted in 16 mL of 0.5 M cupric acetate at 4 °C for 3 h. After the supernatant was collected, 2 mL of the supernatant was incubated with 4 mL of 0.2% (*w/v*) anthrone-sulfuric acid buffer at 100 °C for 5 min. Subsequently, trehalose was determined at 590 nm as previously described [50]. Each treatment had four biological replicates. One sample from individual flask was considered one biological replicate.

### 4.4. Identification of Salt-Stress-Responsive Proteins through 2-DE and MALDI-TOF/TOF MS Analysis

2-DE combined with MALDI-TOF/TOF MS was conducted to investigate the protein profiles of RJS9-2 in response to salt stress. After the growth of RJS9-2 in 100 mL of liquid medium in the flasks with 0.0017 M (CK) or 0.3 M NaCl at 28 °C for 6 days, 40 mL samples of cell cultures were centrifuged at 5000× *g* for 10 min at 4 °C. The precipitated protein pellets were collected and washed four times with 10 mL of PBS buffer containing 100 mM NaCl, 50 mM K_2_HPO_4_, and 50 mM KH_2_PO_4_ (pH 6.7). The remaining pellets were vacuum-dried and dissolved in lysis buffer containing 8 M urea, 2 M thiourea, 0.5% (*v/v*) Triton-100, and 1% (*v*/*v*) β-mercaptoethanol. The protein concentrations were measured according to a 2-D Quant Kit (GE Healthcare, Piscataway, NJ, USA). Then, 80 μg of protein was incubated into an aqueous solution containing 7 M urea, 2 M thiourea, 0.5% (*v/v*) Triton-100, 3% (*w/v*) 3-[(3-cholamidopropyl) dimethylammonio]-1-propanesulfonate (CHAPS), and 1% (*v/v*) immobilized pH gradient (IPG) buffer (pH 4–7). The protein of each sample was separately loaded onto IPG strips (pH 4–7 linear gradient, 11 cm) for isoelectric focusing using an Ettan IPGphor3 system (GE Healthcare, Piscataway, NJ, USA). An Ettan DALTsix electrophoresis unit (GE Healthcare, Piscataway, NJ, USA) was used to perform the second dimension of electrophoresis. Three biological replicates were used for each experiment. An individual flask was considered one biological replicate. Delta 2D software (GE Healthcare, Piscataway, NJ, USA) was used to analyze and identify differentially expressed protein spots. Protein spots that were detected in all three replicates and with an average abundance change of more than 1.5-fold between the control (0.0017 M NaCl) and salt stress (0.3 M NaCl) treatments were selected for MALDI-TOF/TOF MS analysis. An UltrafleXtreme TOF/TOF mass spectrometer (Bruker Daltonics, Karlsruhe, Germany) was used for identification of peptides. The MASCOT (version 2.3, Matrix Science, London, UK) search engine was run against the National Center for Biotechnology Information (Available online: http://www.ncbi.nlm.nih.gov/) database for protein identification according to published methods [51]. A protein score higher than the significance score was considered a successful identification of a protein (Table S1). The KEGG website (Available online: http://www.genome.jp/kegg/) was used to predict the functions of the differentially expressed proteins.

### 4.5. Analysis of Plant Nodulation and Symbiotic Performance under Salt Stress

*S. guianensis* Aubl. genotype Reyan 2 was used. The seeds were soaked in 80 °C for 3 min and germinated for 3 days as described by Chen et al. [21]. The seedlings were transferred to hydroponics box with 10 L of 1/2 Hoagland’s nutrient solution (pH 5.8) and then grown in the greenhouse with temperature ranged from 27 to 35 °C. After 7 days of growth, the seedlings were separately inoculated with the *Bradyrhizobium* strains PN13-3 and RJS9-2. After 30 min of inoculation, all of the seedlings were transferred into 10 L of fresh low-N Hoagland’s nutrient solution containing 50 μM NH_4_NO_3_. After 14 days of growth, the seedlings were separately transferred into low-N Hoagland’s nutrient solution without or with 0.3 M NaCl treatments. The shoots, roots, and nodules were harvested separately after 40 days of NaCl treatments. All of the experiments had three biological replicates. An individual hydroponics box contained three seedlings was regarded as one biological replicate. The N concentrations were measured using the semi-micro Kjedahl procedure with a nitrogen analyzer (Kjedahl 2300; FOSS, Hoganas, Sweden) according to Qin et al. [1].

### 4.6. Statistical Analysis

Microsoft Excel 2003 was used to calculate the mean and standard error. All of the data were analyzed by Duncan’s test or Student’s *t*-test at 0.05 probability level using SAS software (SAS Institute, Cary, NC, USA, v9).

## 5. Conclusions

In conclusion, this study suggests that the high salt tolerance of RJS9-2 was probably associated with IAA production, osmoprotectant biosynthesis, and regulation of specific metabolic pathways. The specific proteins identified here as important in promoting rhizobial growth may contribute to selecting high salt-tolerant strains under salt stress. This study lays the foundation for future studies of proteins that could potentially be employed as new markers or candidate gene resources introduced into bacteria, improving the tolerance of *Bradyrhizobium* strains to severe stress conditions. With the development of biotechnology, a fuller understanding of mechanisms underlying the adaptation of rhizobia to environmental stresses will be obtained.

## Figures and Tables

**Table 1:** Effects of different NaCl levels on the growth of the four tested *Bradyrhizobium* strains.

Strains	Replicates	NaCl (M)						
		CK	0.1	0.2	0.3	0.35	0.4	0.5
RJS9-2	I	++++	++++	++++	++++	+++	+	+
	II	++++	++++	++++	+++	+++	++	−
	III	++++	++++	++++	+++	+++	+	−
	IV	++++	++++	++++	+++	+++	++	+
BS1-1	I	++++	+	+	−	−	−	−
	II	++++	+	−	+	−	−	−
	III	++++	++	+	−	−	−	−
	IV	++++	++	+	−	−	−	−
PN13-3	I	++++	+	+	−	−	−	−
	II	++++	+	++	−	−	−	−
	III	++++	+	+	−	−	−	−
	IV	++++	++	+	−	−	−	−
LZ3-2	I	++++	+	−	−	−	−	−
	II	++++	+	+	−	−	−	−
	III	++++	+	−	−	−	−	−
	IV	++++	+	+	−	−	−	−

The four tested strains were separately inoculated onto solid medium supplied with 0.0017, 0.1, 0.2, 0.3, 0.35, 0.4, or 0.5 M NaCl. Among the different NaCl concentrations, 0.0017 M NaCl was set as the control (CK). All of the plates were incubated in an incubator at 28 °C in the dark. The growth of the four tested strains was recorded after 6 days of NaCl treatment. ++++: No inhibition by salt stress. +++: 30% growth inhibition by salt stress. ++: 60% growth inhibition by salt stress. +: 90% growth inhibition by salt stress. −: No growth under salt stress.

**Table 2:** Identification of salt stress regulated proteins through MALDI TOF/TOF MS analysis.

Spot Number ^a^	Accession Number	Homology Protein	Species	Putative Function	kDa	p*I*	Score	Coverage (%)	Fold Change ^b^
**Enhanced**									
1	WP_006612358	Carbon monoxide dehydrogenase	*Bradyrhizobium* sp. ORS 375	Carbon metabolism	15.40	6.66	89.4	43.92%	6.26 ± 0.29
2	WP_007615200	Superoxide dismutase	*Bradyrhizobium* sp. WSM471	Stress response	22.63	7.02	230	68.18%	4.79 ± 0.14
3	WP_006609834	ABC transporter	*Bradyrhizobium* sp. ORS 285	Transporter	30.81	10.01	73.7	43.10%	4.99 ± 0.21
4	WP_008542552	DNA-binding response regulator	*Bradyrhizobium japonicum* USDA 6	Signal transduction	26.65	5.94	138	42.92%	Induced
**Repressed**									
5	WP_014491960	Zinc protease	*Bradyrhizobium japonicum* USDA 6	Protein metabolism	50.66	5.54	207	28.29%	1.87 ± 0.18
6	WP_014491960	Zinc protease	*Bradyrhizobium japonicum* USDA 6	Protein metabolism	50.66	5.54	222	28.73%	Supressed
7	WP_011087628	Elongation factor Ts	*Bradyrhizobium diazoefficiens* JCM 10833	Protein metabolism	32.17	6.54	111	43.65%	2.88 ± 0.23
8	WP_011926260	30S ribosomal protein S6	*Bradyrhizobium* sp. ORS278	Protein metabolism	17.41	5.01	124	58.17%	3.91 ± 0.18
9	WP_011083272	ATP synthase subunit beta	*Bradyrhizobium diazoefficiens* JCM 10833	Energy metabolism	50.99	4.91	383	59.54%	4.41 ± 0.21
10	WP_007590612	Enolase	*Bradyrhizobium* sp. WSM1253	Energy metabolism	45.36	4.84	271	34.19%	1.77 ± 0.25
11	WP_028155417	β-ketoacyl-ACP synthase I	*Bradyrhizobium japonicum* USDA 6	Other metabolism	42.82	6.17	79.5	24.07%	1.54 ± 0.1
12	WP_014496130	Demethylmenaquinone methyltransferase	*Bradyrhizobium japonicum* USDA 6	Other metabolism	25.90	5.89	383	41.00%	1.54 ± 0.14
13	WP_011090572	Branched-chain amino acid ABC transporter substrate-binding protein	*Bradyrhizobium diazoefficiens* JCM 10833	Transporter	40.84	7.77	151	36.03%	3.80 ± 0.00
14	WP_011088132	DNA-directed RNA polymerease subnit alpha	*Bradyrhizobium diazoefficiens* JCM 10833	Signal transduction	38.03	4.63	306	44.61%	1.82 ± 0.13

^a^ Spot number corresponds to the spot number in Figure 5. ^b^ Data in the column represent the mean of three biological replicates with standard error. An individual flask was considered one biological replicate.

## Supplementary Materials

Supplementary materials can be found at www.mdpi.com/1422-0067/18/8/1625/s1.
