# Supplementary material for: High Salt Tolerance of a Bradyrhizobium Strain and Its Promotion of the Growth of Stylosanthes guianensis
Source: Int J Mol Sci. 2017 Jul 28;18(8):1625. doi: 10.3390/ijms18081625 (PMC5578016; doi:10.3390/ijms18081625)
Supplement: Supplementary file 1 [file ijms-18-01625-s001.pdf]

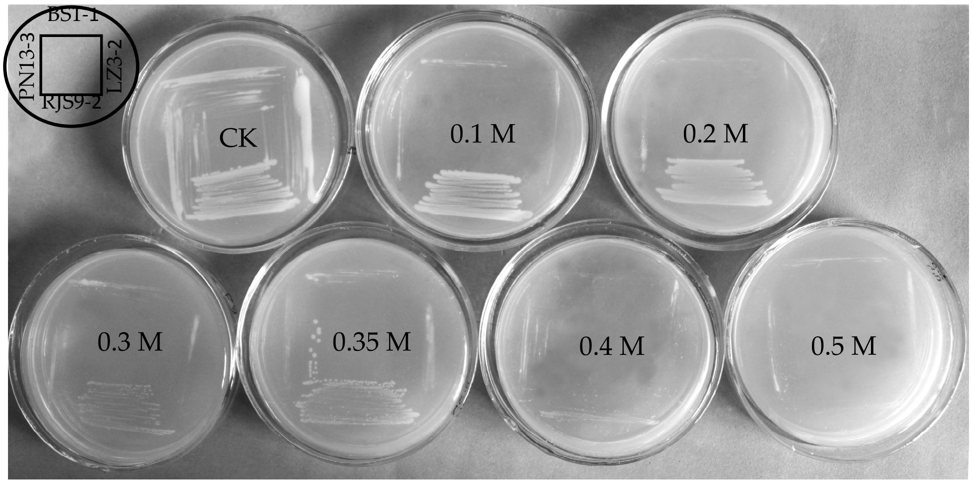

Figure S1. Growth performances of four *Bradyrhizobium* strains under different NaCl treatments. A single clone of the four strains was inoculated onto solid YMA medium supplied with 0.0017, 0.1, 0.2, 0.3, 0.35, 0.4 or 0.5 M NaCl. Among the different NaCl concentrations, 0.0017 M NaCl was set as the control (CK). All of the plates were incubated at 28 °C in the dark. The growth performances of the four tested strains were photographed after 6 d of NaCl treatment. Schematic diagram represents the site of the four tested *Bradyrhizobium* strains in the plate.

Table S1. Details for all MALDI-TOF/TOF successfully identified proteins in RJS9-2 regulated by NaCl.

Spot NO.	Accession number	Protein name	Species	MW (kDa)	Protein score	Sequence coverage (%)	Significance score	No. of matched petides	Start sequence	End sequence	Peptide sequence	Modifications
1	WP_006612358	Carbon monoxide dehydrogenase	*Bradyrhizobium* sp. ORS 375	15.40	89.4	43.92%	63	7	134	144	LADEFFANFAK	-
									78	91	ISGEGEGGVAGFAK	-
									133	144	KLADEFFANFAK	-
									2	15	AMTMTGEVQLAAPR	-
									2	15	AMTMTGEVQLAAPR	Oxidation (M)@2;Oxidation (M)@4
									30	47	ACIPGCEELEKTEDGGFR	Carbamidomethyl (C)@2;Carbamidomethyl (C)@6
									1	21	MAMTMTGEVQLAAPREAVWAK	Oxidation (M)@1;Deamidated (NQ)@10
2	WP_007615200	Superoxide dismutase	*Bradyrhizobium* sp. WSM471	22.63	230	68.18%	63	10	174	179	RPDYLK	-
									45	51	GTEWEGK	-
									52	58	SLEEIVK	-
									101	110	INEDLGGFEK	-
									100	110	KINEDLGGFEK	-
									31	44	HHQAYVTNGNNALK	Deamidated (NQ)@8
									180	197	AFVENLVNWEYVESLFDK	-
									113	133	TDFQAAGVGQFGSGWCWLQVK	Carbamidomethyl (C)@16
									1	21	MTFTLPPLPYAYDALGQFMSK	Oxidation (M)@1;Deamidated (NQ)@17
									142	171	TPNGENPLVHGATPILGCDVWEHSYYIDYR	Carbamidomethyl (C)@18
3	WP_006609834	ABC transporter	*Bradyrhizobium* sp.ORS 285	30.81	73.7	13.10%	63	11	138	146	LQDVTEAIR	Deamidated (NQ)@2
									155	164	VVADNQDAVK	Deamidated (NQ)@5;Deamidated (NQ)@6
									169	179	NLEVFTQSLAR	-
									213	225	DGGELFLTVKSIR	-
									155	168	VVADNQDAVKSSIK	Deamidated (NQ)@5;Deamidated (NQ)@6
									198	212	ADTLMLGLNTLAGGK	Oxidation (M)@5
									184	197	IDGVMSRVDGIMGK	Oxidation (M)@5
									165	179	SSIKNLEVFTQSLAR	Deamidated (NQ)@5;Deamidated (NQ)@11
									191	212	VDGIMGKADTLMLGLNTLAGGK	Deamidated (NQ)@16
									42	63	VVFEGPAAGLRNGGSVNFNGIR	Deamidated (NQ)@12;Deamidated (NQ)@17
									263	289	VLFGASNSSPSNAQASQPAPAPSTSGR	Deamidated (NQ)@7;Deamidated (NQ)@12;Deamidated (NQ)@14
4	WP_008542552	DNA-binding response regulator	*adyrhizobium japonicum* USDA	26.65	138	42.92%	63	9	93	100	MGADDFIR	Oxidation (M)@1
									198	205	TIDSHIKR	-
									58	66	MDGMETLRR	Oxidation (M)@1;Oxidation (M)@4
									1	11	MPTIALVDDDR	-
									43	54	TTQPDLAILDIK	-
									28	42	IMTYTDGASALDGFR	Oxidation (M)@2
									181	197	NALMDAAYDDQVYVDDR	Oxidation (M)@4
									212	229	VVDNEFEMIETLYGVGYR	Oxidation (M)@8
									179	197	SRNALMDAAYDDQVYVDDR	Oxidation (M)@6
5	WP_014491960	Zinc protease	*adyrhizobium japonicum* USDA	50.66	207	28.29%	63	10	204	211	EDALAFYR	-
									433	441	TVAQKWLEK	Deamidated (NQ)@4
									155	164	DVVLEEYNMR	Oxidation (M)@9
									254	265	VRPQEPEPAAPR	-
									93	104	HPVGEFSQTVLR	-
									239	252	NFGAIPAQPAIPAR	-
									127	139	EQLPTMMEFEADR	Oxidation (M)@6;Oxidation (M)@7
									140	154	MTGLILKDENVLPER	Oxidation (M)@1
									383	401	TQLIAEAIYAQDNQAVLAR	-
									105	123	VGGNENASTSVDYTNYYQR	-
6	WP_014491960	Zinc protease	*adyrhizobium japonicum* USDA	50.66	222	28.73%	63	11	204	211	EDALAFYR	-
									423	432	IRAVTAEQVR	-
									155	164	DVVLEEYNMR	Oxidation (M)@9
									254	265	VRPQEPEPAAPR	-
									93	104	HPVGEFSQTVLR	-
									239	252	NFGAIPAQPAIPAR	-
									253	265	RVRPQEPEPAAPR	-
									127	139	EQLPTMMEFEADR	Oxidation (M)@6;Oxidation (M)@7
									140	154	MTGLILKDENVLPER	Oxidation (M)@1
									383	401	TQLIAEAIYAQDNQAVLAR	-
									105	123	VGGNENASTSVDYTNYYQR	-
7	WP_011087628	Elongation factor Ts	*yrhizobium diazoefficiens* JCM 1	32.17	111	43.65%	63	8	177	185	ADELAALGR	-
									86	94	NGQFQGLVK	Deamidated (NQ)@1
									71	85	GVVVEVNSETDFVAR	-
									244	258	EVCLLEQAFIHDTGK	Carbamidomethyl (C)@3
									165	185	MGVIVALESPGKADELAALGR	Oxidation (M)@1
									283	306	YALGEGIEKQESDFAAEVAAASGR	-
									140	164	AASLEVSQGVVSHYVHGAVIDGAGR	-
									186	210	QIAMHVAAANPLALDPSGLDPAVVK	Gln->pyro-Glu (N-term Q)@N_term;Oxidation (M)@4
8	WP_011926260	30S ribosomal protein S6	*Bradyrhizobium* sp. ORS278	17.41	124	58.17%	63	9	125	133	DGGGFRGDR	-
									122	130	GERDGGGFR	-
									2	11	PLYEHVFLAR	-
									93	105	VEELEEGPSAMMR	Oxidation (M)@11;Oxidation (M)@12
									88	105	YLSVRVEELEEGPSAMMR	Oxidation (M)@16;Oxidation (M)@17
									58	77	AHFVLLNIDAPSAAIAEIEI	-
									57	77	KAHFVLLNIDAPSAAIAEIEI	-
									58	80	AHFVLLNIDAPSAAIAEIERQEI	-
									12	36	QDASTQQVEELTTQMTGIVEGLGGK	Gln->pyro-Glu (N-term Q)@N_term;Deamidated (NQ)@1;Deamidated (NQ)@6;Deamidated (NQ)@7
9	WP_011083272	ATP synthase subunit beta	*yrhizobium diazoefficiens* JCM 1	50.99	383	59.54%	63	23	372	379	QVQQVLQR	Gln->pyro-Glu (N-term Q)@N_term
									79	88	VPVGEGTLGR	-
									372	379	QVQQVLQR	-
									372	379	QVQQVLQR	Deamidated (NQ)@1
									430	438	FVDLADTIK	-
									134	143	VVDLLAPYAK	-
									55	66	TIAMDTTEGLVR	Oxidation (M)@4
									232	244	VALTGLTIAEDFR	-
									260	273	FTQAGSEVSALLGR	-
									342	355	GIYPAVDPLDSTSR	-
									187	199	EGNDLYHEFIESK	-
									40	54	LVLEVAQHLGESTVR	-
									215	229	CALVFGQMNEPPGAR	Carbamidomethyl (C)@1;Oxidation (M)@8
									89	104	IINVIGEPIDEAGPVK	-
									356	371	MLSPLVVGEEHYAVAR	Oxidation (M)@1
									245	259	DKGQDVLFFVDNIFR	-
									185	199	TREGNDLYHEFIESK	-
									274	294	IPSAVGYQPTLATDMGALQER	Deamidated (NQ)@8;Deamidated (NQ)@19
									67	88	GQEVTDTGSPIRVPVGEGTLGR	-
									274	294	IPSAVGYQPTLATDMGALQER	Oxidation (M)@15
									448	468	YDHLPEAAFYMVGTIEEAVEK	Oxidation (M)@11
									110	133	AIHQEAPTYTDQSTEAEILVTGIK	-
									382	405	ALQDIIAILGMDELSEEDKLTVAR	Oxidation (M)@11
10	WP_007590612	Enolase	*Bradyrhizobium* sp. WSM1253	45.36	271	34.19%	63	14	271	278	YLADLVSR	-
									254	261	YVYEGEGK	-
									2	10	TAIIDIIGR	-
									181	190	YGAEVFHTLK	-
									251	261	DGKYVYEGEGK	-
									121	132	AAANSLDMPLYR	Deamidated (NQ)@4
									121	132	AAANSLDMPLYR	Oxidation (M)@8
									307	320	CQLVGDDLFVTNVK	Carbamidomethyl (C)@1
									35	52	AAVPSGASTGAHEAVELR	-
									307	321	CQLVGDDLFVTNVKR	Carbamidomethyl (C)@1
									17	34	GNPTVEVDVVLEDGALGR	-
									35	56	AAVPSGASTGAHEAVELRDGDK	-
									279	297	YPIVTIEDGMSEDDMDGWK	Oxidation (M)@10;Oxidation (M)@15
									367	388	SGETEDSTIADLAVATNCGQIK	Carbamidomethyl (C)@18
11	WP_028155417	Beta-ketoacyl-ACP synthase I	*adyrhizobium japonicum* USDA	42.82	79.5	24.07%	63	6	107	116	TIVESADITR	-
									32	41	AEKYAELGFR	-
									94	106	TGIIMGSGGPSAR	Oxidation (M)@5
									205	220	YNDTPATASRPYDVNR	-
									221	241	DGFVIAGGAGVLVLEELEHAK	-
									247	273	IYGEIVGYGATSDGYDMVAPSGEGAER	Oxidation (M)@17
12	WP_014496130	ethylmenaquinone methyltransferas	*adyrhizobium japonicum* USDA	25.9	383	41.00%	63	11	211	219	ETPILEIAR	-
									200	209	LPEAAELCGR	Carbamidomethyl (C)@8
									210	219	RETPILEIAR	-
									124	137	ALGCLGVITDGSIR	Carbamidomethyl (C)@4
									67	80	SVLKSSLSAEEQSK	Deamidated (NQ)@12
									83	96	IEYYEYVGTGHGPR	-
									82	96	RIEYYEYVGTGHGPR	-
									45	61	QLVCPFPDLPPIVGYAR	Gln->pyro-Glu (N-term Q)@N_term;Carbamidomethyl (C)@4
									45	61	QLVCPFPDLPPIVGYAR	Carbamidomethyl (C)@4
									20	36	YDTPTICNAMEIVAPER	Carbamidomethyl (C)@7;Oxidation (M)@10
									20	37	YDTPTICNAMEIVAPERR	Carbamidomethyl (C)@7;Oxidation (M)@10
13	WP_011090572	ino acid ABC transporter substrate-b	*yrhizobium diazoefficiens* JCM 1	40.84	151	36.03%	63	11	325	334	TAKPGTPEFR	-
									193	206	FARPDTSVTGQVLK	-
									346	359	ELAASQGVYNFTEK	-
									178	192	AQAVPMGMTIVDEER	Oxidation (M)@6;Oxidation (M)@8
									302	316	SQFAGHSYDAFEILK	-
									66	83	VIVLDDGGDPTTATTNAR	-
									346	361	ELAASQGVYNFTEKDR	-
									302	317	SQFAGHSYDAFEILKR	-
									235	253	GYQGLIYQTHGAASMDFIR	Oxidation (M)@15
									235	257	GYQGLIYQTHGAASMDFIRIAGK	-
									207	232	LVAANPDAILVGASGTAAALPQTELR	-
14	WP_011088132	directed RNA polymerease subnit al	*yrhizobium diazoefficiens* JCM 1	38.03	306	44.61%	63	16	263	271	VDELELSVR	-
									42	52	GFGQTLGNALR	-
									42	52	GFGQTLGNALR	Deamidated (NQ)@4
									262	271	KVDELELSVR	-
									31	41	FATIVAEPLER	-
									84	94	EDVTDIVLNIK	Deamidated (NQ)@9
									10	19	NWQELIRPNK	Deamidated (NQ)@1
									278	290	NDNIVYIGDLVQK	-
									227	242	ILQDQLNVFVNFEEPR	-
									227	242	ILQDQLNVFVNFEEPR	Deamidated (NQ)@3;Deamidated (NQ)@5
									244	261	EVAQEIIPDLAFNPAFLK	-
									227	243	ILQDQLNVFVNFEEPRK	Deamidated (NQ)@3
									244	262	EVAQEIIPDLAFNPAFLKK	-
									165	185	NRPEDAPIGLIPVDSLYSPVR	-
									205	226	LTMTIETNGAISPDDSVAYAAR	Oxidation (M)@3;Deamidated (NQ)@8
									196	226	EGQILDYDKLTMTIETNGAISPDDSVAYAAR	Deamidated (NQ)@3;Oxidation (M)@12
